# Follicular Helper T Cells in the Immunopathogenesis of SARS-CoV-2 Infection

**DOI:** 10.3389/fimmu.2021.731100

**Published:** 2021-09-16

**Authors:** Dawei Cui, Yuan Tang, Qi Jiang, Daixi Jiang, Yun Zhang, Yan Lv, Dandan Xu, Jian Wu, Jue Xie, Chengping Wen, Liwei Lu

**Affiliations:** ^1^Department of Blood Transfusion, The First Affiliated Hospital, Zhejiang University School of Medicine, Hangzhou, China; ^2^Department of Pathology and Shenzhen Institute of Research and Innovation, The University of Hong Kong, Hong Kong, Hong Kong, SAR China; ^3^Chongqing International Institute for Immunology, Chongqing, China; ^4^Department of Blood Transfusion, Shaoxing People’s Hospital (Shaoxing Hospital, Zhejiang University School of Medicine), Shaoxing, China; ^5^State Key Laboratory for Diagnosis and Treatment of Infectious Diseases, National Clinical Research Center for Infectious Diseases, Collaborative Innovation Center for Diagnosis and Treatment of Infectious Diseases, The First Affiliated Hospital, Zhejiang University School of Medicine, Hangzhou, China; ^6^School of Basic Medical Science, Zhejiang Chinese Medical University, Hangzhou, China

**Keywords:** COVID-19, SARS-CoV-2, Tfh cells, B cells, neutralizing antibody

## Abstract

Coronavirus disease 2019 (COVID-19), caused by the novel severe acute respiratory syndrome coronavirus 2 (SARS-CoV-2), is a serious infectious disease that has led to a global pandemic with high morbidity and mortality. High-affinity neutralizing antibody is important for controlling infection, which is closely regulated by follicular helper T (Tfh) cells. Tfh cells play a central role in promoting germinal center reactions and driving cognate B cell differentiation for antibody secretion. Available studies indicate a close relationship between virus-specific Tfh cell-mediated immunity and SARS-CoV-2 infection progression. Although several lines of evidence have suggested that Tfh cells contribute to the control of SARS-CoV-2 infection by eliciting neutralizing antibody productions, further studies are needed to elucidate Tfh-mediated effector mechanisms in anti-SARS-CoV-2 immunity. Here, we summarize the functional features and roles of virus-specific Tfh cells in the immunopathogenesis of SARS-CoV-2 infection and in COVID-19 vaccines, and highlight the potential of targeting Tfh cells as therapeutic strategy against SARS-CoV-2 infection.

## Introduction

Severe acute respiratory syndrome coronavirus 2 (SARS-CoV-2), an emerging and acute novel coronavirus mainly transmitted *via* the respiratory tract, has rapidly caused pandemic-level cases of coronavirus disease 2019 (COVID-19), which has a high morbidity and mortality worldwide (1–5). Globally, as of 22 June 2021, there have been 178,503,429 confirmed cases of COVID-19, including 3,872,457 deaths from 195 countries and 28 regions according to the World Health Organization (WHO) report (6). SARS-CoV-2 is a serious threat to human health and life worldwide.

**Figure 1:**
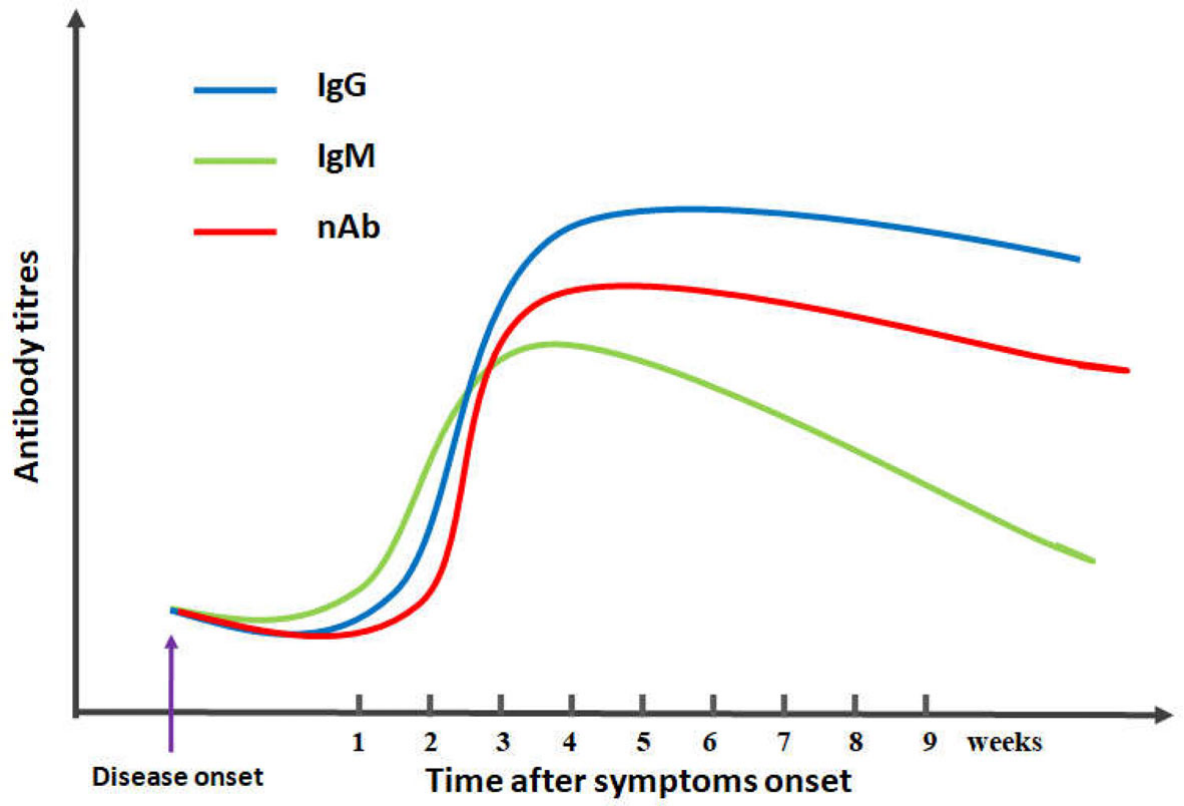
Schematic diagram of antibody kinetics in COVID-19 patients. IgG/IgM/nAb indicates IgG antibodies/IgM antibodies/neutralizing antibodies, respectively.

Humans who are immune-naive to SARS-CoV-2 are considered to be a major factor for the COVID-19 pandemic worldwide, and high-affinity neutralizing antibodies are especially essential for the control and clearance of SARS-CoV-2 infection (7–10). Several studies have reported sustained antibody responses in patients with SARS-CoV-2 infection, in which specific antibody titers are increased along with the progression of infection (11–13) (**Figure 1**). Notably, the titers of specific antibodies against SARS-CoV-2 are usually low in the first week. When the high cumulative seroconversion rate occurs between 2 and 3 weeks after symptom onset, the titers of neutralizing antibodies are significantly decreased in the early convalescent phase, with the titers of neutralizing antibodies not detectable in some patients, which indicate that several weeks may be needed to generate antibodies against SARS-CoV-2 (12–17). These findings suggest that further studies are needed to explore the production and function of neutralizing antibody inSARS-CoV-2 infection.

Antibody responses are closely correlated with CD4^+^T cell subsets that play important roles in the control of viral infections, including T helper (Th) 1 (Th1), Th2, and Th17 cells and follicular helper T (Tfh) cells (18, 19). Among CD4^+^Th cell subsets, naive CD4^+^T cells differentiated into Tfh cells can promote humoral immunity by mediating the interaction between T cells and B cells, which are essential for the control of viral infections and vaccine responses (19–21). Tfh cells, as a novel CD4^+^T cell subset, are characterized by the high expression of CXC chemokine receptor 5 (CXCR5), inducible T cell costimulator (ICOS), programmed cell death protein 1 (PD-1), B-cell lymphoma 6 (Bcl-6), and interleukin-21 (IL-21) in both mice and humans and can usually initiate B cells to differentiate into plasma cells that produce high-affinity antibodies to neutralize the virus, such as lymphocytic choriomeningitis virus (LCMV), influenza virus and hepatitis B virus (22–25). Loss of Tfh cell function can result in primary immunodeficiencies characterized by impaired humoral immunity, including COVID-19 infection, autosomal-dominant hyper IgE caused by *STAT3* deficiency and common variable immunodeficiency (21, 25, 26). However, the roles and function features of Tfh cells in SARS-CoV-2 infection remain largely unclear (19, 20). Here, we will discuss the characteristics and functions of Tfh cells in the immunopathogenesis of SARS-CoV-2 infection and in COVID-19 vaccine responses, as well as their implications in eliciting effective immunity against SARS-CoV-2 infection.

## The Phenotypes and Functions of Tfh Cells

Tfh cells can help B cells generate high-affinity antibodies, long-lived plasma cells, and memory B cells through functional markers (20, 21). The markers of Tfh cells are important to identify Tfh cells and their distinct subsets in the lymphoid tissue and circulation, which commonly include chemokine receptor CXCR5, transcription factor Bcl-6, PD-1, CD40 ligand (CD40L), and ICOS in humans and mice (25, 27–29). Moreover, the phenotypes of Tfh cells are associated with different stages of immune responses (30, 31). In secondary lymphoid organs, naïve CD4^+^T cells are differentiated into Tfh cells with the upregulation of CXCR5 and downregulation of CC-chemokine receptor 7 (CCR7), which are mediated by antigen-specific conventional dendritic cells (DCs) or monocyte-derived DCs (28, 32, 33). The increased CXCR5 and decreased CCR7contribute to the migration of Tfh cells toward CXC-chemokine ligand 13 (CXCL13)-enriched B lymphoid follicles in the germinal center (GC) (28, 34). The specific transcription factor Bcl-6 is selectively expressed in Tfh cells but is highly expressed in CXCR5^hi^CCR7^low/-^Tfh cells in human and mouse GCs (34–37). The IL-21 cytokine is highly and specifically secreted by Tfh cells, which promotes the proliferation of Tfh cells and helps B cell differentiation and antibody secretion, which is characteristic of Tfh cells (38–42). ICOS deficiency significantly reduces GC reactions and Tfh cells in mice and humans, which indicates that ICOS expressed in Tfh cells is essential for the differentiation and maintenance of Tfh cells, GC formation, B cell differentiation and antibody responses (43–45). ICOS, as a key costimulatory molecule, can also induce the secretion of IL-21 in Tfh cells (45–47). HighPD-1 expression on Tfh cells can significantly promote the differentiation and activity of Tfh cells (48–50). Collectively, Tfh cells are commonly identified as having three phenotypes: canonical GC Tfh cells with PD-1^++^ and ICOS^++^Bcl-6^+^CCR7^-^CXCR5^++^CD4^+^T cells, precursor-Tfh (Pre-Tfh) cells characterized as PD-1^+^ICOS^+^Bcl-6^low^CCR7^low^CXCR5^+^CD4^+^T cells, and memory Tfh cells similar to Pre-Tfh cells in lymphoid tissue (36, 50–52). In GC, Tfh cells are responsible for regulating B cell differentiation into memory B cells and plasma cells, controlling the selection of high-affinity antibody production and the development of long-term humoral immunity (53–56).

Circulating Tfh (cTfh) cells in the peripheral blood are usually composed of two distinctive phenotypes: effector memory Tfh cells (PD-1^+^ICOS^+^CCR7^low^BCL-6^-^CXCR5^+^CD4^+^T cells) and central memory Tfh cells (PD-1^-^ICOS^-^CCR7^high^BCL-6^-^CXCR5^+^CD4^+^T cells) (32, 57, 58). Additionally, based on the expression of CXCR3 and CCR6, cTfh cells are further divided into three subsets: Tfh1 (CXCR3^+^CCR6^-^), Tfh2 (CXCR3^-^CCR6^-^), Tfh17 (CXCR3^-^CCR6^+^), and Tfh1/17 (CXCR3^+^CCR6^+^) cells, which share the signature transcription factors and cytokines of Th1 (T-bet and IFN-γ), Th2 (GATA3, IL-4, IL-5 and IL-13), Th17 (RORγt, IL-17 and IL-22) cells, respectively (32, 58, 59). cTfh2 and cTfh17 cells can induce B cell differentiation and antibody secretion and regulate immunoglobulin (Ig) isotype switching. cTfh1 cells are commonly considered not to be a helper for B cells, but ICOS^+^PD-1^high^CCR7^low^cTfh1 cells effectively regulate B cell differentiation and induce antibody responses (59–65). These studies display functionally distinct cTfh cell subsets based on ICOS, PD-1, and CCR7 expression, as well as CXCR3 and CCR6. Moreover, these novel subsets are different from Th1, Th2 and Th17 cells but share some of their characteristics. Additionally, Tfh-like cells have also been identified in non-lymphoid tissues, including the synovium of arthritis, skin and salivary glands of patients, which commonly express low or undetectable CXCR5 and Bcl-6 and high PD-1, ICOS, OX40 and IL-21 compared to Tfh cells in secondary lymphoid organs, which also express tissue-specific chemokine receptors, including CCR2, CCR5, CX3C-chemokinereceptor 1 (CX3CR1) and CXCR4 (52, 66–71). Recently, Tfh13 cells, a novel Tfh cell subset that secretes IL-4 and IL-13, were shown to be responsible for IgE production in human and mouse allergies and to highly express the transcription factors Bcl-6 and GATA3 (72–74). Current studies indicate that distinct phenotypes of Tfh cells are critical for B cell differentiation and high-affinity antibody production (**Table 1**). Interestingly, follicular regulatory T (Tfr) cells are considered a subset of Foxp3^+^Treg cells in the GC that are initiated from Foxp3^+/-^ precursors but not from Tfh cells (75–78). Tfr cells share canonical Tfh cell molecules, including CXCR5, Bcl-6, PD-1 and ICOS, as well as Treg cell molecules, including CD25, Foxp3, Blimp-1 and CTLA-4 (79–82). Importantly, Tfr cells, similar to Treg cells, play a critical role in immunosuppression, rather than Tfh cells, which can limit GC responses and suppress the activation of Tfh cells and B cells within GCs through inhibitory molecules, including CTLA-4, PD-1, IL-10 and TGF-β secretion. The balance of Tfh/Tfr cells is essential to maintain immune homeostasis and mediate humoral immunity (63, 66, 82–85).

**Table 1:** Phenotypes of Tfh cell subsets in blood and lymphoid tissues.

Location	Cell subsets	Phenotypic markers	References
**Blood**			
	Central memory Tfh cells	PD-1^-^ICOS^-^CCR7^high^Bcl-6^-^Blimp-1^-^CXCR5^+^	(32, 57, 58)
	Effector memory Tfh cells	CD40L^+^/PD-1^+^/ICOS^+^CCR7^low^Bcl-6^-^Blimp-1^-^CXCR5^+^	
	cTfh1 cells	IFN-γ^+^Bcl-6^-^Blimp-1^-^CXCR5^+^or PD-1^+^ICOS^+^CCR7^low^CXCR3^+^CCR6^-^Bcl-6^-^Blimp-1^-^CXCR5^+^	(32, 57–59)
	cTfh2 cells	IL-4^+^Bcl-6^-^Blimp-1^-^CXCR5^+^ or CXCR3^-^CCR6^-^Bcl-6^-^Blimp-1^-^CXCR5^+^	
	cTfh17 cells	IL-17A^+^Bcl-6^-^Blimp-1^-^CXCR5^+^or CXCR3^-^CCR6^+^Bcl-6^-^Blimp-1^-^CXCR5^+^	
	cTfh1/17 cells	IFN-γ^+^ IL-17A^+^Bcl-6^-^Blimp-1^-^CXCR5^+^or CXCR3^+^CCR6^+^Bcl-6^-^Blimp-1^-^CXCR5^+^	
	cTfh13 cells	IL-13^hi^IL-4^hi^IL-5^hi^IL-21^low^Bcl-6^+^GATA3^+^CXCR5^+^	(72–74)
**Lymphoid tissues**			
	Pre-Tfh cells	PD-1^+^ICOS^+^CCR7^low^Bcl-6^low^Blimp-1^-^CXCR5^+^	(32, 57, 58)
	GC Tfh cells	PD-1^++^ICOS^++^CCR7^-^Bcl-6^+^Blimp-1^-^CXCR5^++^	
	Memory Tfh cells	PD-1^+^ICOS^+^CCR7^low^Bcl-6^low^Blimp-1^-^CXCR5^+^	

PD-1, programmed cell death protein-1; CCR7, CC-chemokine receptor 7; CXCR3, CXC-chemokine receptor 3; CCR6, CXC-chemokine receptor 6; CXCR5, CXC-chemokine receptor 5; ICOS, inducible T cell co-stimulator.

## The Differentiation of Tfh Cells

Tfh cell differentiation is regulated by multiple complex factors and stages. Naïve CD4^+^T cells are primed by binding their T cell receptors with peptide-loaded major histocompatibility complex (MHC) class II (pMHC-II) on professional antigen-presenting cells (APCs), such as DCs and monocytes. Strong TCR signaling and continuous antigenic stimulation play critical roles in favoring Tfh cell differentiation by upregulating BATF to promote Bcl-6 expression (86–90). The early differentiation of Tfh cells is sufficiently initiated by DCs predominantly localized to T cell zones of lymphoid organs, which are considered Pre-Tfh cells that upregulate Bcl-6 and CXCR5 and repress CCR7 expression, and Bcl6^+^CXCR5^+^Pre-Tfh cells are attracted by the chemokine CXCL13 (CXCR5 ligand) produced within the B cell follicle zones toward the T-B border (36, 64, 91–94). Pre-Tfh cells migrate to the T-B cell border and interact with cognate B cells to further upregulate Bcl-6, CXCR5, ICOS, PD-1 and IL-21 and downregulate CCR7 expression, which further drives GC-Tfh differentiation and maturation and GC formation. These processes also require available costimulatory molecules and cytokines, including ICOS-ICOSL, OX40-OX40L, PD-1-PD-Ll/2, CD40-CD40L, IL-21, IL-6 and IL-12 cytokines (25, 32, 36, 95–102).

The transcription factor Bcl-6 in CD4^+^T cells is mostly essential for Tfh differentiation and function, and loss of Bcl-6 represses Tfh differentiation, GC formation, B cell differentiation and antibody responses (34, 35, 43). Bcl6-expressing Tfh cells are also regulated by multiple transcription factors, including positive inductors such as TCF-1 and LEF-1, BATF, NOTCH1/2, and IRF4 and negative regulators such as Blimp-1, FOXO1 and STAT5 (22, 25, 32, 103–110). Some costimulatory molecules expressed on Tfh cells are considered markers of Tfh cells, including ICOS, OX40, PD-1 and CD40L, which can also induce Tfh cell differentiation and maintenance (32, 111, 112). In GC, B cells highly express costimulatory ligands, including ICOSL, CD80, CD86, PD-L1, and PD-L2, which contribute to the maintenance of Tfh cells, and then Tfh cells also mutually promote B cells to differentiate into plasma cells to further produce specific antibodies that mediate humoral immune responses (113, 114). Bcl-6 induces secretion of the cytokine IL-21, which can promote Tfh cell differentiation by upregulating STAT-1 and STAT-3 signals to further induce Bcl-6 expression, and similarly, the cytokine IL-6 plays a critical role in Tfh cell differentiation by upregulating the STAT1/3-Bcl-6 signal axis (56, 85, 115, 116). In addition, Tfh1 cells are characterized by IL-21 and IFN-γ production, and Tfh1 cell differentiation characterized by increased T-bet and Bcl-6 expression is initiated by phosphorylation of STAT1 and STAT4 in CD4^+^T cells that are induced through IL-12, which is partially inhibited by a high concentration of IL-2 that reduces Bcl-6 expression (85, 115–120). Tfh2 cells are characterized by IL-4 and IL-21 production; Tfh2 cell differentiation is driven by IL-4 but suppressed by IL-6 *via* STAT3 signaling, and IL-4-secreted Tfh2 cells contribute to humoral immunity (85, 121–123). Tfh17 cells are characterized by IL-21 and IL-17 production; Tfh17 cell differentiation is primed by IL-23, IL-21, ICOS, TGF-β and IL-6, which upregulate Bcl-6 and RORγt expression. Consistent with its well established role in driving B cell response during infection, IL-17 secreted by Tfh17 cells can promote interactions of cognate T-B cells in the GC, inducing the formation of spontaneous GC and Ig isotype class-switching (124–127). However, low doses of IL-2, TGF-β and CTLA-4 promote the development of Tfr cells that play critical roles in inhibiting Tfh cell differentiation and GC responses by activating STAT5, Blimp-1, and Bach2 transcription factors in Tfr cells characterized by CXCR5^+^Foxp3^+^CD4^+^T cells (128–133). Tfr cells can inhibit Tfh cell and plasma cell differentiation by inhibitory molecules, including CTLA-4, IL-10 and TGF-β; conversely, Tfh cells also inhibit the expansion of Tfr cells by the IL-21 cytokine (27, 131–139). This suggests that the balance of Tfh and Tfr cells plays a critical role in regulating B cell differentiation and specific antibody production (140).

## Tfh Cells in SARS-CoV-2 Infection and Vaccine

**Figure 2:**
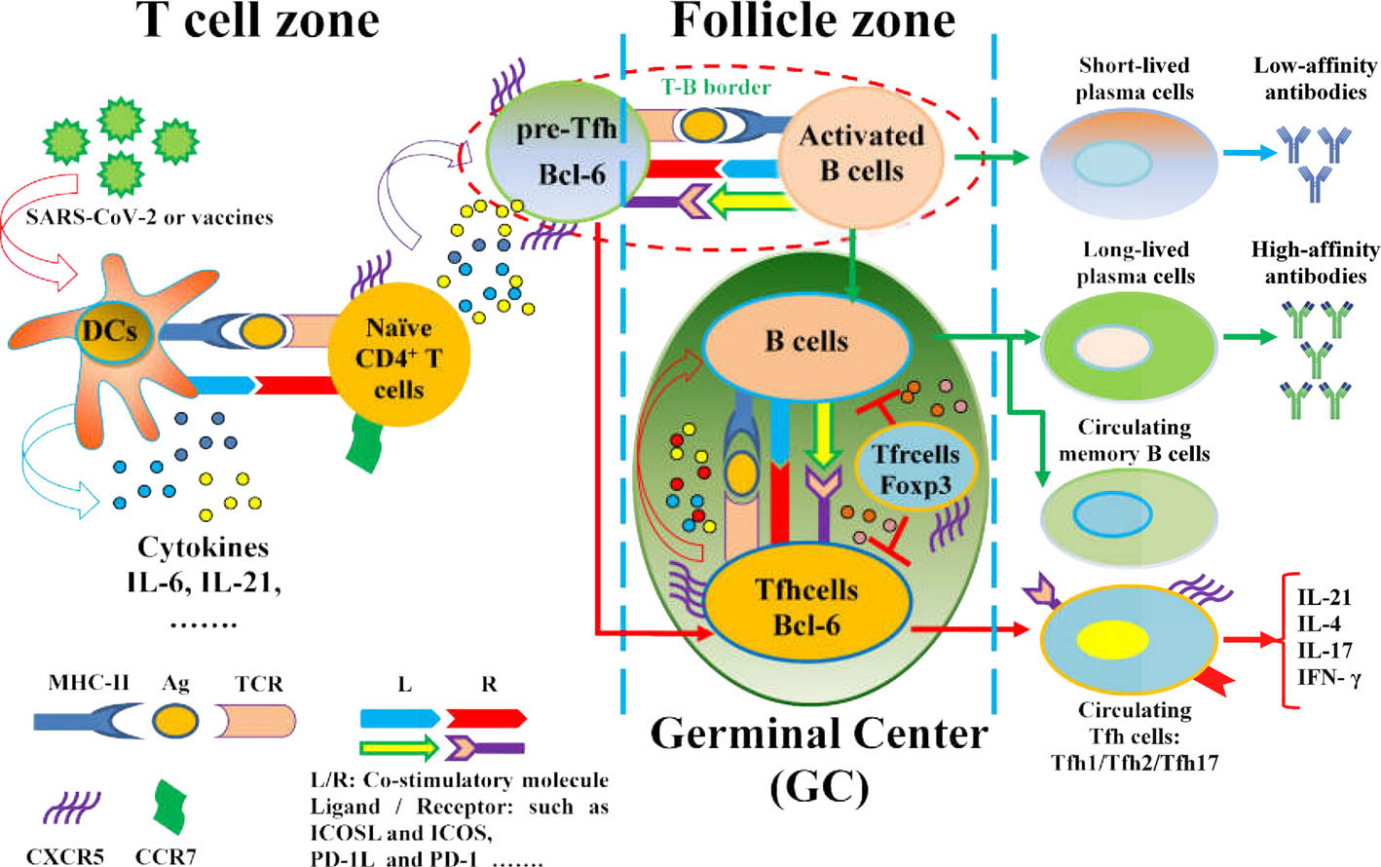
The regulation of Tfh cell differentiation and function in SARS-CoV-2 infection and vaccines. Naïve CD4^+^ T cells are driven by APCs (DCs) upon exposure to SARS-CoV-2 or virus antigens, which are activated toward antigen-specific Pre-Tfh cells with upregulation of CXCR5 and Bcl-6 and downregulation of CCR7 under the interaction of MHC-II molecules on DCs and cognate TCR on CD4^+^T cells, as well as the expression of costimulatory molecules and cytokine production. Pre-Tfh cells interact with activated B cells at the T-B border in the follicle zone, which further differentiate into various Tfh cell subsets that migrate to the GC, where Tfh cells promote B cell differentiation and specific antibody production. However, the loss of GC structures reduces Bcl-6^+^Tfh cells in severe COVID-19 patients. Notably, SARS-CoV-2-specific Tfh cells are expanded in mild and asymptomatic patients with COVID-19.Moreover, vaccines can efficiently induce Tfh cell differentiation, GC formation, and protective antibody responses.

Currently, the SARS-CoV-2 infection pandemic has led to a serious threat to human health worldwide. Neutralizing antibodies of humoral immunity play a critical role in vaccine responses and battles against infectious viruses, including SARS-CoV-2, which is closely associated with Tfh cells differentiation and function (18, 19, 21, 141–144) (**Figure 2**). The role and function of Tfh cells in the control and clearance of SARS-CoV-2 infection and in the development of new vaccines have been investigated.

Previous reports showed that the frequencies of cTfh cells characterized byCXCR5^+^ICOS^+^PD-1^+^progressively increased up to 20 days from the onset of infection in a case with non-severe convalescent COVID-19, in addition to elevated specific plasma SARS-CoV-2-binding IgM and IgG antibodies (145). Single-cell analysis revealed that expanded frequencies of cTfh cells were found in patients with active COVID-19 disease, as well as a high percentage of specific anti-SARS-CoV-2 antibodies, including IgA and IgG (146). The frequencies of spike (S)-specific cTfh cells (CD3^+^CD4^+^CD45RA^–^CXCR5^+^) are consistently elicited after S peptide stimulation in convalescent COVID-19 cases and exhibit a clear phenotypic bias to aCCR6^+^CXCR3^-^cTfh17 cell phenotype; however, neutralizing activity is inversely correlated with S-specific cTfh17 cell frequencies but positively correlated with S-specific cTfh, cTfh1 (CCR6^-^CXCR3^+^) and cTfh2 (CCR6^-^CXCR3^-^) cell frequencies (147). Previous reports suggested that expanded CXCR3^+^cTfh1 cells positively correlated with the neutralizing antibody response against influenza vaccination and live-attenuated yellow fever vaccination (148, 149). A recent study showed that increased frequencies of CCR7^low^PD-1^+^cTfh-effectormemory (em), cTfh1 and cTfh2 cells in CXCR5^+^CD45RA^-^CD25^-^CD4^+^T cells are significantly increased, as well as high IL-1β and TNF-α, and that the frequencies of cTfh1 cells are associated with SARS-CoV-2-specific IgG/IgM antibodies, although CCR7^high^PD-1^-^cTfh-central memory (cm) and cTfh17 cells in CXCR5^+^CD45RA^-^CD25^-^CD4^+^T cells are decreased, as well as cTfr cells in Treg cells in convalescent patients compared to healthy subjects. Moreover, the frequencies of high cTfh-em, low cTfh-cm and cTfr cells are positively correlated with disease severity (150). These observations indicated that cTfh cell phenotypes can induce potent neutralizing responses against SARS-CoV-2 in COVID-19convalescent patients, which will contribute to antibody-based therapeutics and vaccination design for COVID-19.

Additionally, increased frequencies of virus-specific cTfh cells (CD4^+^CXCR5^+^OX40^+^CD40L^+^) were observed in acute and convalescent COVID-19 cases, and the frequencies of both SARS-CoV-2-specific cTfh cells and S-specific CCR6^+^CXCR3^-^cTfh17 cells were closely associated with low disease severity (151). Longitudinal studies on COVID-19 infection and convalescent subjects indicate that the levels of SARS-CoV-2 antibodies are low and insufficient in humoral immunity response, although the underlying mechanism is poorly understood (11–14). The numbers of CD4^+^CXCR5^+^Tfh, ICOS^+^Tfh, Bcl-6^+^Tfh and Bcl-6^+^B cells are decreased in lymph nodes and spleens, which are possibly associated with exclusively abundantTh1 cells, increased Treg cells (but not Tfr cells) and aberrant TNF-α production in COVID-19 lymph nodes in COVID-19 patients, as well as loss of GCs in lymph nodes and spleens from acute and dead COVID-19 patients (26, 152, 153). These data indicated that defective Tfh cell generation and dysregulated humoral immunity provide a possible mechanistic explanation for the limited durability of antibody responses in COVID-19 disease. Furthermore, low frequencies of CD45RA^-^PD-1^+^CXCR5^+^cTfh cells were also observed, but elevated frequencies of activated cTfh (CD38^+^ICOS^+^) cells were positively correlated with anti-SARS-CoV-2 IgM and IgG titers in hospitalized COVID-19 patients (154). These findings indicated that activated cTfh cells may be more reflective of recent antigen encounter and emigration from the GCs. Additionally, a single-cell transcriptomic analysis revealed that increased proportions of cytotoxic cTfh cells in hospitalized COVID-19 patients early in the illness are negatively correlated with the IgG levels of anti-spike protein antibodies to SARS-CoV-2, although the total SARS-reactive cTfh cells show a positive correlation with anti-spike antibody levels in hospitalized COVID-19 patients but not in non-hospitalized COVID-19 patients, which provided insights into cytotoxic cTfh cells in the distinct disease severities of COVID-19 patients (155). Moreover, reduced cTfh and PD-1^+^cTfh and increased exhausted TIM‐3^+^cTfh cell frequencies are significantly observed, but the correlations between cTfh cells and anti-SARS-CoV-2IgM and IgG titers were not analyzed in hospitalized COVID-19 patients (156). These results indicated that cytotoxic cTfh and exhausted cTfh cells may inhibit specific anti-SARS-CoV-2 antibody production, which plays a critical role in severe SARS-CoV-2 infection (157). In a recent cohort study of COVID-19 patients within six months of recovery, the CXCR5^+^CD4^+^cTfh cell frequencies were significantly higher in COVID-19 patients in the long-term clinically recovered (20∼26 weeks) cohort (LCR) than in those in the short-time clinically recovered (4∼9 weeks) cohort (SCR). However, the frequencies of cTfh cells in both the LCR and SCR cohorts were lower than those in the healthy donor cohort (HD). Moreover, three cTfh subsets were similar between the LCR and HD cohorts; cTfh1 cell frequencies in the SCR cohort were shown to be significantly low, but cTfh2 and cTfh17 subsets were found to be high compared with the LCR and HD cohorts (158). Virus-specific Tfh cell frequencies, memory B cell responses, and serum CXCL13 levels were not different between asymptomatic or mild symptomatic COVID-19 patients. In contrast, COVID-19 patients with moderate or severe disease exhibited vigorous virus-specific GC B cell responses and Tfh cell responses. Moreover, potent virus-specific Th1 and CD8^+^T cell responses were observed in asymptomatic or mildly symptomatic patients but not in severely symptomatic patients. These data suggest that asymptomatic and mild patients have weak and transient SARS-CoV-2 antibody responses (159).

During acute COVID-19 infection, expanded activated CD38^+^HLA-DR^+^PD-1^+^ICOS^+^CXCR5^+^CD4^+^cTfh cells, CD38^+^HLA-DR^+^CXCR3^+^cTfh1 cells, and activated CD38^+^HLA-DR^+^Th1 cells emerged, together with cytotoxic CD8^+^T cells. The number of activated cTfh1 cells positively correlated with the levels of RBD- and spike-specific antibodies, including IgG, IgA and IgM isotypes (160). These data indicated that activated cTfh cell responses were associated with robust antibody responses elicited during SARS-CoV-2 infection, which may be valuable as potential biomarkers in vaccine clinical trials. Similarly, CD38^+^HLA-DR^+^cTfh cells, activated CD4^+^T cells and cytotoxic CD8^+^T cells were expanded in COVID-19 patients, and increased CD38^+^HLA-DR^+^cTfh cells indicated a recent antigen encounter and emigration from the GC of the patients (161). The frequencies of PD-1^+^ICOS^+^cTfh, cytotoxic CD4^+^T and exhausted T cells were strongly expanded in COVID-19 patients, particularly in severe patients compared to healthy individuals, which suggested that extensive T cell dysfunction was associated with COVID-19 severity (162). In severe COVID-19 patients, the frequencies of CCR6^+^cTfh cells and CCR4^+^cTfh cells were expanded, but CCR3^+^cTfh cells and Th1 cells were low in severe COVID-19 patients compared to healthy individuals (163). The frequencies of PD-1^+^ICOS^+^cTfh cells, activated cTfh cells and cytotoxic CD8^+^T cells were strongly upregulated in COVID-19 patients, particularly in severe patients compared to healthy donors. Moreover, an increase in CD4^+^CD127^-^CD25^+^Treg cells was found in mild patients, and upregulation of CCR4 in activated CD8^+^T cells indicated enhanced lung homing in severe COVID-19 patients (164). Additionally, in rhesus macaques, SARS-CoV-2 infection induces predominantly GC CXCR3^+^Tfh cells (but not a PD-1^++^Foxp3^+^Tfr cell subset) specific for the SARS-CoV-2 spike and nucleocapsid proteins and produce high titers of antiviral serum IgG and IgM antibodies againstSARS-CoV-2 (165) (**Table 2**). These data indicated that variable Tfh cell subsets dysregulated the humoral immune responses in COVID-19 patients caused by SARS-CoV-2 infection.

**Table 2:** Characteristics and function of Tfh cells in COVID-19 patients.

Severity of disease	Characteristics	Function	Isotype of antibodies	References
Convalescent	CD3^+^CD4^+^CD45RA^-^CXCR5^+^Tfh cells expansion; bias to a CCR6^+^CXCR3^-^cTfh17 cells.	Positively associate with plasma neutralizing activity.	—	(146)
	cTfh-em and cTfh1 cells expansion.	Positively associate with the SARS-CoV-2-specific antibody titers.	IgM	(149)
Mild	CXCR5^+^ICOS^+^PD-1^+^cTfh cells expansion,	Correlate with better clinical outcomes.	IgM, IgG	(144)
	CD45RA^-^PD-1^+^CXCR5^+^cTfh cells reduction, activated cTfh (CD38^+^ICOS^+^) cells expansion.	Positively correlate with anti-SARS-CoV-2IgM and IgG titers.	IgM, IgG	(153)
Moderate	TIM-3^+^Tfh-like cells expansion, CD226^+^Tfh-like cells reduction.	Benefit the maintenance of balanced cellular and humoral immune responses.	—	(155)
Severe	Tem and Tfh-em cells expansion, Tcm, Tfh-cm, and Tfr cells reduction.	cTfh-em cells negatively correlate with recorded PaO_2_/FiO_2_.	IgG, IgA	(149)
	Cytotoxic cTfh cells and cytotoxic T helper cells expansion, Treg cells reduction.	Negatively correlate with antibody levels to SARS-CoV-2spike protein.	—	(154)
	PD-1^+^ICOS^+^CXCR5^+^CD4^+^cTfh cells expansion.	Correlate with robust humoral immunity.	IgG, IgM, and IgA	(159)
	CCR6^+^cTfh cells and CCR4^+^cTfh cells expansion, CCR3^+^cTfh cells and Th1 cells reduction.	Favor the development of the antibody response.	—	(162)

“—” indicates not mentioned; Tfh, follicular helper T cell; cTfh, circulating Tfh cell; cTfh-em, effector-memory-like circulating Tfh cell; Tfh-cm, central-memory-like circulating Tfh cell; Tfr, follicular T regulatory cell; PaO_2_, arterial oxygen tension; FiO_2_, inspiratory oxygen fraction;Treg, regulatory T cells.

The COVID-19 pandemic continues to spread worldwide, and a safe and protective vaccine is urgently needed to effectuate herd protection and control of SARS-CoV-2. Currently, rapid advances have been made in the design and development of SARS-CoV-2 vaccines, such as inactivated vaccines, DNA vaccines, mRNA vaccines and specific SARS-CoV-2 proteins (166). mRNA-1273 vaccine could significantly induce Th1 and interleukin-21-producing CXCR5^+^PD−1^+^ICOS^+^Tfh cell responses, and elicit robust SARS-CoV-2 neutralizing activity, which provided rapid protection in the upper and lower airways from SARS-CoV-2 infection in Rhesus Macaques (167). When compared to SARS-CoV-2 with recombinant SARS-CoV-2 receptor-binding domain (rRBD) formulated with AddaVax (rRBD-AddaVax) protein vaccine, the SARS-CoV-2 mRNA vaccines encoding RBD and full-length spike protein efficiently induce SARS-CoV-2-specific GC B cell and Tfh cell responses, which promoted specific neutralizing antibody production in vaccinated mice. Interestingly, the rRBD-AddaVax vaccine could elicit high frequencies of IL-4^+^ Tfh cells (168). In human vaccination, the BNT162b2 mRNA vaccine for SARS-CoV-2 had significantly elicited AIM^+^CXCR5^+^CD45RA^-^CD3^+^cTfh cell responses, AIM (activation induced marker) cells include CD69^+^OX40^+^ or CD69^+^CD40L^+^ orCD69^+^4-1BB^+^ or OX40^+^4-1BB^+^ or CD40L^+^4-1BB^+^ or CD40L^+^OX40^+^cells, and the frequency of AIM^+^cTfh cells is positively correlated with anti-Spike-specific IgA and IgG antibody titers (169). These findings have indicated that SARS-CoV-2 mRNA vaccines can effectively promote antigen-specific Tfh cell differentiation, B cell responses and the generation of protective antibodies, which are considered as promising candidates for eliciting high-quality adaptive immune responses to control and clear SARS-CoV-2 infection. Additionally, the specific protein vaccines including SARS-CoV-2 subunit vaccine (NVX-CoV2373) with the full-length spike (S) protein, StriFK-FH002C and Spike (S)/receptor binding domain (RBD) protein subunit vaccine significantly induce specific cTfh cell and GC B cell responses, resulting in high neutralizing antibody titers of SARS-CoV-2 (170–172) (**Table 3**). Various clinical trials in humans indicate that inactivated SARS-CoV-2 vaccines can induce satisfactory high neutralizing antibody titers that notably reduce the number of patients with severe COVID-19 (173–176). These data suggested that SARS-CoV-2 vaccines can safely and effectively promote humoral immune responses, enhance neutralizing antibody titers, and reduce the incidence and mortality of critically ill patients.

**Table 3:** Tfh cell responses in various vaccine candidates of SARS-CoV-2.

Vaccine candidates	Phenotypes	Function	Antibody isotypes	References
**mRNA vaccines**				
mRNA-1273	IL-21^+^CXCR5^+^PD−1^+^ICOS^+^Tfh cells expansion.	Induce robust and specific antibody responses including neutralizing antibody.	IgA, IgG	(166)
full SΔ furin mRNA	B220^-^CD4^+^CD44^hi^CD62L^-^CXCR5^+^Bcl-6^+^ Tfh cells, B220^-^CD4^+^CD44^hi^CXCR5^+^PD-1^hi^ IL-21^+^Tfh cells, B220^-^CD4^+^CD44^hi^CXCR5^+^Bcl-6^+^ ICOS^+^Tfh cells B220^-^CD4^+^CD44^hi^CXCR5^+^PD-1^hi^ IFN-γ^+^Tfh cells notable expansion.	Elicit potent SARS-CoV-2-specific GC B responses, induce robust and specific antibody responses including neutralizing antibody.	IgG1, IgG2a, IgG2b,	(167) (167)
RBD mRNA (receptor binding domain, RBD)	B220^-^CD4^+^CD44^hi^CD62L^-^CXCR5^+^Bcl-6^+^ Tfh cells, B220^-^CD4^+^CD44^hi^CXCR5^+^PD-1^hi^ IL-21^+^Tfh cells, B220^-^CD4^+^CD44^hi^CXCR5^+^Bcl-6^+^ ICOS^+^Tfh cells, B220^-^CD4^+^CD44^hi^CXCR5^+^PD-1^hi^ IFN-γ^+^Tfh cells notable expansion	Elicit potent SARS-CoV-2-specific GC B responses, induce robust and specific antibody responses including neutralizing antibody.	IgG1, IgG2a, IgG2b,	
BNT162b2 mRNA vaccine	AIM^+^CXCR5^+^CD45RA^-^CD3^+^cTfh cells expansion, AIM cells include CD69^+^OX40^+^ or CD69^+^CD40L^+^ orCD69^+^4-1BB^+^ or OX40^+^4-1BB^+^ or CD40L^+^4-1BB^+^ or CD40L^+^OX40^+^	Positively correlate with anti-spike-specific IgA and IgG titers.	IgA, IgG	(168)
**Protein vaccines**				
rRBD-AddaVax	B220^-^CD4^+^CD44^hi^CD62L^-^CXCR5^+^Bcl-6^+^ Tfh cells, B220^-^CD4^+^CD44^hi^CXCR5^+^PD-1^hi^ IL-21^+^Tfh cells B220^-^CD4^+^CD44^hi^CXCR5^+^PD-1^hi^ IL-4^+^Tfh cells slight expansion	Delay to elicit potent SARS-CoV-2-specific GC B responses, induce robust and specific antibody responses including neutralizing antibody.	IgG1,	(167)
NVX-CoV2373	CXCR5^+^PD-1^+^CD4^+^Tfh cells expansion	Induce specific antibody responses including neutralizing antibody.	IgG	(169)
Spike (S) and receptor binding domain (RBD) protein subunit vaccine	CXCR5^++^BCL-6^+^CD4^+^CD3^+^B220^-^Tfh cells expansion	Induce specific antibody responses including neutralizing antibody.	IgG	(170)
StriFK-FH002C	PD-1^+^CXCR5^+^CD4^+^Tfh cells expansion	Induce specific antibody responses including neutralizing antibody.	IgG, IgG1, IgG2a, IgG2b	(171)

Tfh, follicular helper T cell; cTfh, circulating Tfh cell.

## Conclusions

Tfh cells and associated molecules play a critical role in the development of viral infection, and Tfh cell subsets are required for high-quality neutralizing antibodies from B cells to control and clear viruses including SARS-CoV-2, which can effectively promote humoral immune responses. Emerging evidence indicates that functional characterization of Tfh cells and their subsets will provide novel insights into improved vaccine design and therapeutic strategies to prevent and control various viral infections including SARS-CoV-2 infection.

## Author Contributions

DC drafted the manuscript and designed the figures and tables. FX, QJ, DJ, YL, DX, and JW revised the manuscript. DC, JX, CW, and LL conceived the topic and revised the manuscript. All authors contributed to the article and approved the submitted version.

## Funding

This work was supported by the National Natural Science Foundation of China (Grant Nos. 81871709, 81971994, 82071817 and 91846103), Funding for Chongqing International Institute for Immunology (2020YJC10), Hong Kong Research Grants Council General Research Fund (17113319) and Theme-Based Research Scheme (T12-703/19R), Zhejiang Provincial Key Research and Development Program (Grant No. 2020C03032).

## Conflict of Interest

The authors declare that the research was conducted in the absence of any commercial or financial relationships that could be construed as a potential conflict of interest.

## Publisher’s Note

All claims expressed in this article are solely those of the authors and do not necessarily represent those of their affiliated organizations, or those of the publisher, the editors and the reviewers. Any product that may be evaluated in this article, or claim that may be made by its manufacturer, is not guaranteed or endorsed by the publisher.
